# Genotypic Variation in Cultivated and Wild Sorghum Genotypes in Response to *Striga hermonthica* Infestation

**DOI:** 10.3389/fpls.2021.671984

**Published:** 2021-07-08

**Authors:** Nicoleta Muchira, Kahiu Ngugi, Lydia N. Wamalwa, Millicent Avosa, Wiliter Chepkorir, Eric Manyasa, Desterio Nyamongo, Damaris A. Odeny

**Affiliations:** ^1^Department of Plant Science and Crop Protection, University of Nairobi, Nairobi, Kenya; ^2^International Crops Research Institute for the Semi-Arid Tropics-Kenya, Nairobi, Kenya; ^3^Kenya Agricultural and Livestock Research Organization, Genetic Resources Research Institute, Kikuyu, Kenya

**Keywords:** witchweed, residual heterosis, DArT-seq, CWR, pre-breeding, *Striga hermonthica*

## Abstract

*Striga hermonthica* is the most important parasitic weed in sub-Saharan Africa and remains one of the most devastating biotic factors affecting sorghum production in the western regions of Kenya. Farmers have traditionally managed *Striga* using cultural methods, but the most effective and practical solution to poor smallholder farmers is to develop *Striga*-resistant varieties. This study was undertaken with the aim of identifying new sources of resistance to *Striga* in comparison with the conventional sources as standard checks. We evaluated 64 sorghum genotypes consisting of wild relatives, landraces, improved varieties, and fourth filial generation (F_4_) progenies in both a field trial and a pot trial. Data were collected for days to 50% flowering (DTF), dry panicle weight (DPW, g), plant height (PH, cm), yield (YLD, t ha^−1^), 100-grain weight (HGW, g), overall disease score (ODS), overall pest score (OPS), area under *Striga* number progress curve (ASNPC), maximum above-ground *Striga* (NS_max_), and number of *Striga*-forming capsules (NSFC) at relevant stages. Genetic diversity and hybridity confirmation was determined using Diversity Arrays Technology sequencing (DArT-seq). Residual heterosis for HGW and NS_max_ was calculated as the percent increase or decrease in performance of F_4_ crossover midparent (MP). The top 10 best yielding genotypes were predominantly F_4_ crosses in both experiments, all of which yielded better than resistant checks, except FRAMIDA in the field trial and HAKIKA in the pot trial. Five F_4_ progenies (ICSVIII IN × E36-1, LANDIWHITE × B35, B35 × E36-1, F6YQ212 × B35, and ICSVIII IN × LODOKA) recorded some of the highest HGW in both trials revealing their stability in good performance. Three genotypes (F6YQ212, GBK045827, and F6YQ212xB35) and one check (SRN39) were among the most resistant to *Striga* in both trials. SNPs generated from DArT-seq grouped the genotypes into three major clusters, with all resistant checks grouping in the same cluster except N13. We identified more resistant and high-yielding genotypes than the conventional checks, especially among the F_4_ crosses, which should be promoted for adoption by farmers. Future studies will need to look for more diverse sources of *Striga* resistance and pyramid different mechanisms of resistance into farmer-preferred varieties to enhance the durability of *Striga* resistance in the fields of farmers.

## Introduction

Sorghum [*Sorghum bicolor* (L.) Moench] is a diploid (2*n* = 2*x* = 20) cereal grass of the Gramineae family native to Africa (Doggett, 1988). It is the fifth most important cereal globally (Kiprotich et al., 2015) and a major staple food for more than 300 million inhabitants of Africa (Kidanemaryam et al., 2018). In Kenya, sorghum is ranked second after maize (*Zea mays* L.) in tonnage and production area, which is approximately 117,000 ha (FAOSTAT, 2016). Drought stress and poor soil fertility are the major abiotic factors affecting sorghum production in semi-arid areas (Ejeta and Knoll, 2007). Biotic stresses include diseases such as anthracnose (*Colletotrichum graminicola*) (Marley et al., 2005), leaf blight (*Exserohilum turcicum*) (Beshir et al., 2015), and the parasitic weed (*Striga hermonthica*).

The genus *Striga* comprises over 30 species, of which *S. hermonthica*, also known as the purple witchweed, is the most important in sub-Saharan Africa. *S. hermonthica* parasitizes several major cereal crops including maize, sorghum, rice (*Oryza sativa* L.), finger millet [*Eleusine coracana* (L.) Gaertn.], and pearl millet [*Pennisetum glaucum* (L.) R. Br.]. It remains one of the most devastating biotic factors affecting sorghum production in the western regions of Kenya (Khan et al., 2006) often characterized by low fertility and high moisture stress. The weed germinates on stimulation by a strigolactone (Bouwmeester et al., 2019; Aliche et al., 2020) induced by the host, or in some cases, non-host plants. The germinated *Striga* then attaches to the roots of the host plants, using a special invasive organ, the haustorium (Yoshida and Shirasu, 2009). The haustorium enables uptake of water and nutrients from the host plants for the growth and development of *Striga*, as well as the introduction of phytotoxins to the host (Van Hast et al., 2000). Consequently, the growth and development of the host plants become severely affected resulting in yield losses of up to 100% (Kim et al., 2002; Ejeta, 2007).

An adult *Striga* plant can produce up to 100,000 tiny seeds that can survive in the soil for 20 years or more (Pieterse and Pesch, 1983; Gurney et al., 2005), making it extremely difficult to control. Previous studies have reported *Striga* seed and plant densities in western Kenya at about 1,188 seeds per mature *Striga* seed capsule (Van Delft et al., 1997) and about 14 plants per m^2^ (MacOpiyo et al., 2010), respectively. In Kenya, the three crops most devastated by *S. hermonthica* are maize, finger millet, and sorghum. Traditionally, farmers have managed *Striga* in sorghum fields using cultural and mechanical methods including hand weeding (Frost, 1994), intercropping (Aasha et al., 2017), and crop rotations (Oswald and Ransom, 2001) with edible legumes such as common bean (*Phaseolus vulgaris* L.), pigeon pea [*Cajanus cajan* (L.) Millsp.], and mung bean [*Vigna radiata* (L.) R. Wilczek].

Pathogenic isolates of *Fusarium oxysporum* f. sp. *Strigae* have been reported to be effective as bioherbicides, especially when integrated with other control practices (Rebeka et al., 2013). The push–pull technology that involves the intercropping of cereals with a trap crop (pull), usually Napier grass (*Pennisetum purpureum*), and a forage legume, usually desmodium (*Desmodium* spp.), as a push crop (Khan et al., 2011) has resulted in low adoption due to the lack of use for desmodium by farmers. “Suicidal death” of *Striga*, which is achieved by inducing the germination of *Striga* by non-host legumes, has been employed in the reduction of *Striga* seed banks (Rubiales and Fernández-Aparicio, 2012). Chemical control has been tested in maize (Menkir et al., 2010) and sorghum (Bouréma et al., 2005; Dembélé et al., 2005; Tuinstra et al., 2009), but it is not environmentally friendly besides being unaffordable for the average sorghum farmer in Kenya. The most effective and practical solution for the smallholder sorghum farmers is to develop *Striga*-resistant sorghum varieties.

Sorghum germplasm screening against *Striga* is the first step toward the identification of *Striga*-resistant genotypes. Resistance has been reported among cultivated sorghum varieties including N13 (Haussmann et al., 2004), SRN39, FRAMIDA, and IS9830 (Ezeaku, 2005; Rodenburg et al., 2005). The resistance mechanism in N13 is a hypersensitive reaction characterized by thickening of the cell wall and silica deposition that limits xylem–xylem connection with the host plants (Maiti et al., 1984). SRN39, on the other hand, is known to harbor pre-attachment resistance that results in the production of a low germination stimulant, orobanchol (Satish et al., 2012; Mohemed et al., 2016). N13 and SRN39 have been used extensively as sources of resistance (Hess and Ejeta, 1992; Ngugi et al., 2015; Yohannes et al., 2015; Ali et al., 2016), and the quantitative trait loci (QTLs) responsible for resistance have been mapped (Haussmann et al., 2004; Satish et al., 2012). The outcrossing nature of *Striga* that results in different ecotypes with mixed responses to different genotypes (Fantaye, 2018) would require the pyramiding of multiple alleles from diverse sources into farmer-preferred varieties if the resistance were to be durable. Crop wild relatives (CWRs) and landraces of sorghum have been reported with significantly higher resistance to *Striga* than N13 and SRN39 (Mbuvi et al., 2017; Mallu et al., 2021). Such reports provide strong justification for more screening of CWRs and landraces toward the identification of additional sources of resistance to *Striga*.

Recommended methodologies for effective field screening include inoculation with *Striga* seeds, appropriate experimental designs with sufficient replications, quantitative data scoring, and inclusion of susceptible and resistant checks at regular intervals (Haussmann et al., 2000b; Rodenburg et al., 2005). A quantitative measure such as “area under *Striga* number progress curve” (ASNPC) alongside *Striga* count, *Striga* vigor, and grain yield has been used in past studies (Haussmann et al., 2012; Abate et al., 2017) with great success. The aim of this study was to screen for novel sources of resistance to *Striga* using both wild and landrace sorghum accessions, improved sorghum varieties, selected F_4_ progenies, and known *Striga*-resistant sources, such as N13, FRAMIDA, HAKIKA, IS9830, and SRN39, as checks.

## Materials and Methods

### Plant Material and Experimental Design

Sixty-four sorghum genotypes (Table 1) consisting of 17 wild relatives, 8 landraces, 13 improved varieties (high yielding), and 26 F_4_ progenies of selected parents were used in this study. The parental lines of the crosses included five improved varieties (B35, E36-1, MACIA, ICSVIII IN, and F6YQ212) and five landraces (LODOKA, OKABIR, IBUSAR, AKUOR-ACHOT, and LANDIWHITE). Successful crosses were selected morphologically at F_1_ and advanced to F_4_ using the bulk-population method.

**Table 1:** Sorghum genotypes used, their sources, and classification.

**Genotype**	**Source**	**Classification**	***Species***
1. GBK 044058	GeRRI	Wild	*Sorghum* sp.
2. GBK 044336	GeRRI	Wild	*Sorghum* sp.
3. GBK 048922	GeRRI	Wild	*Sorghum* sp.
4. GBK 047293	GeRRI	Wild	*Sorghum arundinaceum* (Desv.) Stapf
5. GBK 048916	GeRRI	Wild	*Sorghum* sp.
6. GBK 016085	GeRRI	Wild	*Sorghum arundinaceum* (Desv.) Stapf
7. GBK 048917	GeRRI	Wild	*Sorghum* sp.
8. GBK 016114	GeRRI	Wild	*Sorghum sudanense* (Piper) Stapf
9. GBK 044063	GeRRI	Wild	*Sorghum* sp.
10. GBK 048156	GeRRI	Wild	*Sorghum arundinaceum* (Desv.) Stapf
11. GBK 016109	GeRRI	Wild	*Sorghum arundinaceum* (Desv.) Stapf
12. GBK 044120	GeRRI	Wild	*Sorghum* sp.
13. GBK 040577	GeRRI	Wild	*Sorghum arundinaceum* (Desv.) Stapf
14. GBK 048921	GeRRI	Wild	*Sorghum* sp.
15. GBK 044448	GeRRI	Wild	*Sorghum* sp.
16. GBK 045827	GeRRI	Wild	*Sorghum purpureosericeum* (Hochst. ex A. Rich.) Asch. and Schweinf.
17. GBK 048152	GeRRI	Wild	*Sorghum arundinaceum* (Desv.) Stapf
18. GBK 044065	GeRRI	Landrace	*Sorghum* sp.
19. GBK 043565	GeRRI	Landrace	*Sorghum arundinaceum (Desv.)* Stapf
20. GBK 044054	GeRRI	Landrace	*Sorghum almum Parodi*
21. OKABIR	ICRISAT	Landrace	*Sorghum bicolor*
22. IS9830^*^	ICRISAT	Improved variety	*Sorghum bicolor*
23. HAKIKA^*^	ICRISAT	Improved variety	*Sorghum bicolor*
24. AKUOR-ACHOT	ICRISAT	Landrace	*Sorghum bicolor*
25. LODOKA^†^	ICRISAT	Landrace	*Sorghum bicolor*
26. E36-1^†^	ICRISAT	Improved variety	*Sorghum bicolor*
27. B35^†^	ICRISAT	Improved variety	*Sorghum bicolor*
28. N13^*^	ICRISAT	Landrace	*Sorghum bicolor*
29. SRN39^*^	ICRISAT	Improved variety	*Sorghum bicolor*
30. KARIMTAMA-1	ICRISAT	Improved variety	*Sorghum bicolor*
31. GADAM	ICRISAT	Improved variety	*Sorghum bicolor*
32. F6YQ212	ICRISAT	Improved variety	*Sorghum bicolor*
33. MACIA	ICRISAT	Improved variety	*Sorghum bicolor*
34. FRAMIDA^*^	ICRISAT	Improved variety	*Sorghum bicolor*
35. KAT/ELM/2016 PL82 KM32-2	ICRISAT	Improved variety	*Sorghum bicolor*
36. KAT/ELM/2016 PL1 SD15	ICRISAT	Improved variety	*Sorghum bicolor*
38. ICSVIII_IN	ICRISAT	Improved variety	*Sorghum bicolor*
39. OKABIR × AKUOR-ACHOT	UON	F4 Population	*Sorghum bicolor*
40. AKUOR-ACHOT × ICSVIII_IN	UON	F4 Population	*Sorghum bicolor*
41. B35 × AKUOR-ACHOT	UON	F4 Population	*Sorghum bicolor*
42. B35 × E36-1	UON	F4 Population	*Sorghum bicolor*
43. B35 × F6YQ212	UON	F4 Population	*Sorghum bicolor*
44. B35 × ICSVIII_IN	UON	F4 Population	*Sorghum bicolor*
45. B35 × LANDIWHITE	UON	F4 Population	*Sorghum bicolor*
46. B35 × LODOKA	UON	F4 Population	*Sorghum bicolor*
47. E36-1 × MACIA	UON	F4 Population	*Sorghum bicolor*
48. F6YQ212 × B35	UON	F4 Population	*Sorghum bicolor*
49. F6YQ212 × LODOKA	UON	F4 Population	*Sorghum bicolor*
50. IBUSAR × E36-1	UON	F4 Population	*Sorghum bicolor*
51. IBUSAR × LANDIWHITE	UON	F4 Population	*Sorghum bicolor*
52. IBUSAR × ICSVIII_IN	UON	F4 Population	*Sorghum bicolor*
53. ICSVIII_IN × B35	UON	F4 Population	*Sorghum bicolor*
54. ICSVIII_IN × E36- 1	UON	F4 Population	*Sorghum bicolor*
55. ICSVIII_IN × LANDIWHITE	UON	F4 Population	*Sorghum bicolor*
56. ICSVIII_IN × LODOKA	UON	F4 Population	*Sorghum bicolor*
57. ICSVIII_IN × MACIA	UON	F4 Population	*Sorghum bicolor*
58. LANDIWHITE × B35	UON	F4 Population	*Sorghum bicolor*
59. LANDIWHITE × MACIA	UON	F4 Population	*Sorghum bicolor*
60. LODOKA × ICSVIII_IN	UON	F4 Population	*Sorghum bicolor*
61. LODOKA × LANDIWHITE	UON	F4 Population	*Sorghum bicolor*
62. LODOKA × OKABIR	UON	F4 Population	*Sorghum bicolor*
63. OKABIR × B35	UON	F4 Population	*Sorghum bicolor*
64. OKABIR × ICSVIII_IN	UON	F4 Population	*Sorghum bicolor*

*GeRRI, Genetic Resources Research Institute-Kenya; F_4_, fourth filial generation; ICRISAT, International Crops Research Institute for the Semi-Arid Tropics; UON, University of Nairobi*.

* *Striga-resistant checks*.

† *Staygreen source*.

The field and pot trials were set up during the long rainy season of 2019 at a *S. hermonthica* hotspot in Alupe, Busia County, Western Kenya. Alupe is located on 34° 07′ 28.6″ E and 00°30′ 10.1″ N with an annual rainfall range of 1,100–1,450 mm and daily mean temperatures of 24°C. The area has a bimodal rainfall pattern of long and short rains. Both experiments were laid out in a square lattice design with three replications, each block consisting of eight plots. The field experiment was planted in a *Striga*-infested field with a spacing of 75 cm between rows and 20 cm between plants in the row. Each row contained 21 plants. *Striga* inoculum was prepared by mixing 5 kg of sand with 15 g of *Striga* seeds that had been harvested from the same location in the previous season. A supplemental *Striga* inoculum of 15 g was spread along each row during planting to improve the consistency of *Striga* seed load across the plot. Phosphorus (P) was applied at the rate of 90 Kg ha^−1^ after thinning, while nitrogen (N) was applied at the rate of 92 kg ha^−1^ when the plants were 45–50 cm tall, which was around 30 days after germination. Insect pests, especially fall armyworm (*Spodoptera frugiperda*) and cutworms (*Agrotis* spp., *Spodoptera* spp., and *Schizonycha* spp.), were controlled using Voliam Targo® SC (Syngenta Crop Protection AG, Switzerland) containing active ingredients such as chlorantraniliprole and abamectin. The field experiment was purely rainfed.

For the pot experiment, pots of 30 cm diameter were filled with 20 kg of *Striga*-free soil obtained from *Striga*-free field. Each pot contained four plants and was used to represent a plot. The pot experiment was set up in the field alongside the field experiment and was not under any shelter. The pot experiment was rainfed as much as possible but due to the restricted pot size and high levels of evaporation from the pots, watering was done only when the plants were close to the permanent wilting point. *Striga* inoculation was done by adding 5 g of *Striga* inoculum to each pot. The application of fertilizer and insect pest control was carried out as already described.

### Agronomic and *Striga* Data Collection

The data on agronomic traits were collected from six randomly selected plants from each plot, while the data on *Striga* response traits were collected per plot. The agronomic data were collected for days to 50% flowering (DTF), dry panicle weight (DPW, g), plant height (PH, cm), yield (YLD, t ha^−1^), and 100-grain weight (HGW, g), at relevant stages as per the recommendations of the International Board for Plant Genetic Resources (IBPGR) and the International Crops Research Institute for the Semi-Arid Tropics (ICRISAT) (1993). The date of first *Striga* emergence was recorded followed by *Striga* count at 2-week intervals until maturity. The number of *Striga* plants forming capsules (NSFC) per plot was recorded at 105 days after sowing. We used the overall disease score (ODS) and the overall pest score (OPS) on a scale of 1–9 to account for any diseases and pests observed, ranging from leaf blight (*Helminthosporium turcicum*), ladder leaf spot (*Cercospora fuscimaculans*), zonate leaf spot (*Gloeocercospora sorghi*), anthracnose (*C. graminicola*), spider mites (*Oligonychus pratensis* and *Tetranychus urticae*), sorghum midge (*Contarinia sorghicola*), and sorghum shoot fly (*Atherigona soccata*).

### ANOVA and *Striga* Data Analysis

The maximum above-ground *Striga* (NS_max_) was calculated as suggested by Rodenburg et al. (2006). The ASNPC was calculated according to the formula suggested by Haussman et al. (2000) as follows:

$$\begin{array}{l} ASNPC=\overset{n-1}{\underset{i=0}{\sum }}\left[\frac{Y_{i}+Y_{\left(i+1\right)}}{2}\right]\left(t_{\left(i+1\right)}-t_{i}\right) \end{array}$$

where *n* is the number of *Striga* assessment dates, *Y*_*i*_ is the *Striga* count at the *i*th assessment date, and *t*_*i*_ is the number of days after sowing at the *i*th assessment date.

ANOVA and means for quantitative traits were generated in the alpha lattice design using Genstat software version 19.1 (VSN International, 2017). Treatment means were compared using Fisher's protected least significant differences at *P* ≤ 0.05. The estimates of phenotypic and genotypic variance and the genotypic and phenotypic coefficients of variation were performed based on the formula proposed by Syukur et al. (2012).

Genotypic variance:

$$\begin{array}{l} \sigma_{g}^{2}=\frac{MS_{g}-MS_{e}}{r} \end{array}$$

Phenotypic variance:

$$\begin{array}{l} \sigma_{p}^{2}=\sigma_{g}^{2}+\sigma_{e}^{2} \end{array}$$

where $\sigma_{g}^{2}$ = genotypic variance, $\sigma_{p}^{2}$ = phenotypic variance, $\sigma_{e}^{2}$ = environmental variance (i.e., error mean square from the ANOVA), *MS*_*g*_ = mean square of genotypes, *MS*_*e*_ = error mean square, and *r* = number of replications.

Genotypic coefficient of variation:

$$\begin{array}{l} \left[GCV\right]=\left[\frac{\left\{\sqrt{\sigma_{g}^{2}}\right\}}{x}\right]\times 100 \end{array}$$

Phenotypic coefficient of variation:

$$\begin{array}{l} \left[PCV\right]=\left[\frac{\left\{\sqrt{{\sigma^{2}}_{p}}\right\}}{x}\right]\times 100 \end{array}$$

where $\sigma_{g}^{2}$ = genotypic variance, $\sigma_{P}^{2}$ = phenotypic variance, and *x* is grand mean of a character.

Simple linear correlation coefficients were calculated to understand the relationship among the studied agronomic traits for each trial according to the formula given below:

$$\begin{array}{l} P_{X,Y}=\frac{cov\left(x,y\right)}{\sigma_{x}\sigma_{Y}} \end{array}$$

where *cov* is the covariance, σ_*x*_ is the SD of *x*, and σ_*y*_ is the SD of *Y*.

Phenotypic correlations across the field and pot trials were estimated, and correlation plots were drawn by using R Version 4.0.4 according to the formula described by (Hallauer et al., 2010):

$$\begin{array}{l} r_{xy}=\frac{\sum \left(x_{i}-\bar{x}\right)\left(y_{i}-ȳ\right)}{\sqrt{{\sum {\left(x_{i}-\bar{x}\right)}^{2}\sum \left(y_{i}-ȳ\right)}^{2}}} \end{array}$$

where *r*_*x,y*_ is the correlation coefficient of each trait between the two sites, *x* (field trial), and *y* (pot trial); $\bar{x}$and ȳ are the means of the values of each of the traits in the field and pot trials, respectively. The significance of linear relationships in phenotypic correlation coefficients across the two trials was compared with *r*-coefficient values and the associated degrees of freedom (*n* = 2), at the probability levels of *P* = 0.05, 0.01, and 0.001.

### Heritability Estimates

Estimations of broad-sense heritability (*H*^2^) for all traits were calculated based on parental and family means, respectively, according to the formula described by Allard (1960):

$$\begin{array}{l} H^{2}bs=\left[\frac{\sigma_{g}^{2}}{\sigma_{p}^{2}}\right]\times 100 \end{array}$$

*H*^2^*bs* = heritability in broad sense, $\sigma_{g}^{2}$ = genotypic variance, and $\sigma_{p}^{2}$ = phenotypic variance. *H*^2^ scores were classified according to Robinson et al. (1949) as follows: 0–30% = low, 30–60% = moderate, and >60% = high.

### Genotyping, Genetic Relatedness, and Confirmation of True Crosses

Molecular data of all the 37 parental genotypes in Table 1, which consisted of 17 wild accessions, 8 landraces, and 12 improved varieties, were generated for the genetic diversity analysis. DNA extraction, genotyping, and filtering of raw SNPs were performed as described by Ochieng et al. (2020). A neighbor-joining (NJ) dendrogram was generated using the Trait Analysis by aSSociation, Evolution, and Linkage (TASSEL) software version 5.2.67 (Bradbury et al., 2007). To undertake the hybridity confirmation, DNA was extracted from additional 115 F_4_ progenies that were representative of all the crosses, bringing the total number of individuals genotyped to 153. The filtered SNP variant call file for each of the genotyped F_4_ progenies was parsed through the GenosToABHPlugin in TASSEL 5.2.67 alongside the corresponding parents to obtain informative biallelic SNPs in the ABH format (i.e., female parent alleles as “A,” male parent allele as “B,” and heterozygotes as “H”).

### Comparing the Agronomic Performance of Parents and Progenies Under *Striga* Field Trial Conditions

Crosses involving parents that failed to germinate were excluded from the analysis. The percentage increase or decrease in the performance of F_4_ crossover midparent (MP) was calculated to observe the residual heterotic effects for HGW and NS_max_. The average F_4_-values per cross were used for the estimation of residual heterosis expressed in percentage over MP as described by Turner (1953).

where,

MP-value = (P1 + P2)/2
Residual heterosis = [(F_4_ – MP)/MP] × 100

## Results

### Field Trial and Pot-Screening Trial

All the trait means from the field and pot trials are provided in Supplementary Table 1. The two environments had an effect on all traits except PH (Table 2) and OPS (Table 3). Significant differences (*P* ≤ 0.001) were observed in the agronomic performance of genotypes for all traits across the field and pot trials (Table 2). There were also significant differences (*P* ≤ 0.001) in the performance of the parental lines when compared with the progenies, except for DTF in the field trial and PH in the pot trial (Table 2). We observed more consistency across replications in the pot trial for all agronomic traits than in the field trial, where differences across replications were observed for HGW and PH (Table 2).

**Table 2:** Mean squares of agronomic traits measured under field trial and pot trial.

	**SOV**	**HGW**	**YLD**	**DTF**	**PH**
Combined	Environment	21.65^***^	2,779.93^***^	4,555.74^***^	545.20ns
Field trial	Reps	2.21^*^	1.05ns	81.84ns	2,355.90^***^
	Genotype	2.34^***^	4.40^***^	319.67^***^	6,421.40^***^
	Parents (Par)	2.04^***^	2.69^***^	213.02^***^	5,566.20^***^
	Progenies (Pro)	2.31^***^	5.41^***^	465.04^***^	6,655.70^***^
	Par × Pro	13.16^***^	42.86^***^	98.2ns	27,930^***^
	Residual	0.75	0.76	49.87	366.62
Pot trial	Reps	2.1421ns	32.81ns	21.87ns	205.80ns
	Genotype	1.99^***^	164.89^***^	177.24^***^	6,192.20^***^
	Parents (Par)	3.92^***^	118.66^***^	140.09^***^	6,860^***^
	Progenies (Pro)	1.67ns	154.39^***^	228.61^***^	5,356.80^***^
	Par × Pro	17.84^***^	2,081.54^***^	335.70^***^	592ns
	Residual	1.30	40.69	45.17	575.75

*HGW, 100-grain weight; YLD, yield; DTF, days to 50% flowering; PH, plant height*.

* *Significant at P < 0.05*.

*** *Significant at P < 0.001*.

*ns, non-significant*.

**Table 3:** Mean squares of *Striga-*, disease-, and pest-related traits measured under field trial and pot trial.

	**SOV**	**ASNPC**	**NS_**max**_**	**NSFC**	**ODS**	**OPS**
Combined	Environment	35,611,065^***^	71,590.40^***^	10,788.95^***^	1.80^**^	4.9515ns
Field trial	Reps	2,289,177ns	3,319^*^	384.8^*^	0.43ns	2.59^***^
	Genotype	7,332,224^***^	4,620^***^	570^***^	3.68^***^	3.96^***^
	Parents (Par)	7,863,173^***^	5,402^***^	742.2^***^	3.56^***^	2.89^***^
	Progenies (Pro)	6,203,111^***^	3,676^***^	340.4^***^	3.69^***^	5.30^***^
	Par × Pro	292,494^***^	880ns	318.7ns	7.41^***^	4.44^***^
	Residual	1,860,949	1,333.39	135.32	0.58	0.54
Pot trial	Reps	491,477^***^	349.13^***^	2.59ns	0.35ns	4.10^**^
	Genotype	333,365^***^	73.39^***^	8.64^***^	5.28^***^	2.59^***^
	Parents (Par)	448,828^***^	83.86^***^	10.02^***^	4.53^***^	2.57^***^
	Progenies (Pro)	169,309^***^	57.10^***^	6.73^**^	4.36^***^	2.27^***^
	Par × Pro	162,659^*^	93.81^***^	5.54ns	58.79^***^	10.98^***^
	Residual	88,840	11.18	3.96	1.12	1.04

*ASNPC, area under Striga number progress curve; NS_max_, maximum above-ground Striga; NSFC, number of Striga-forming capsules; ODS, overall disease score; OPS, overall pest score*.

* *Significant at P < 0.05*.

** *Significant at P < 0.01*.

*** *Significant at P < 0.001*.

*ns, non-significant*.

**Figure 1:**
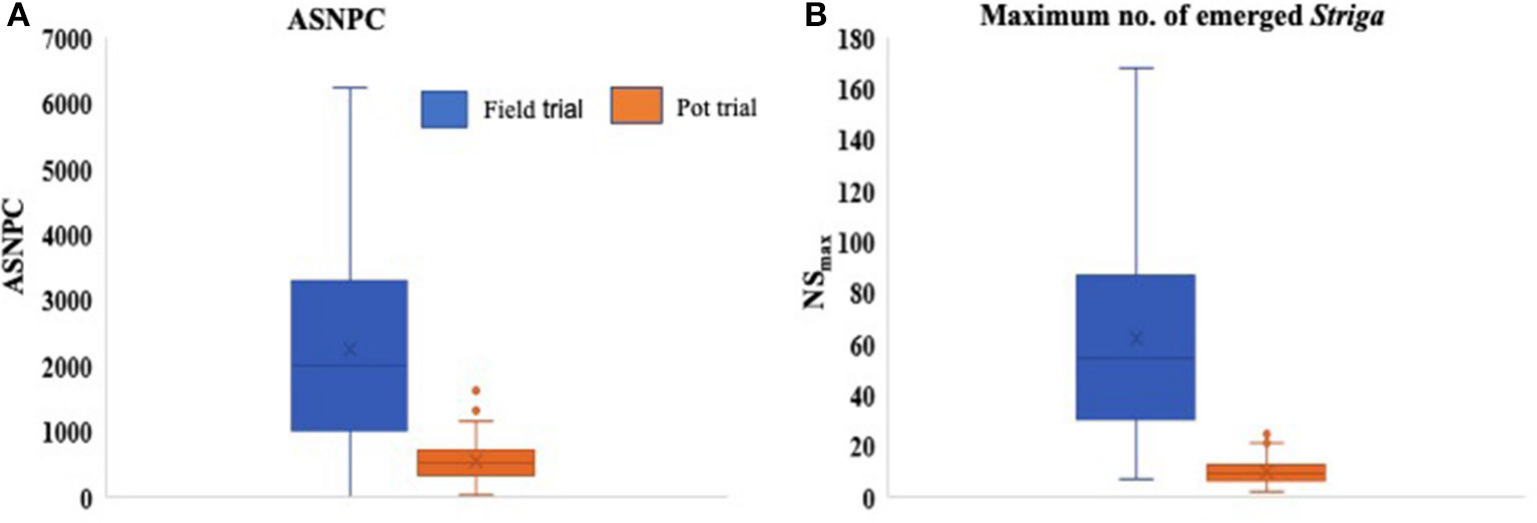
Box plots comparing the overall means of area under *Striga* number progress curve (ASNPC) **(A)** and maximum above-ground *Striga* (NS_max_) **(B)** of genotypes in the field and pot trials.

Significant differences (*P* < 0.001) were observed between genotypes for all *Striga*-related traits, as well as for ODS and OPS (Table 3). The performance of parents against their progenies also revealed significant differences (*P* < 0.001) for ASNPC, ODS, and OPS. More consistency across replications was observed in the field trial for ASNPC and ODS and in the pot trial for NSFC and ODS (Table 3). Yield-related traits (i.e., YLD and HGW) were consistently higher in the pot trial than in the field trial, while *Striga*-related traits (i.e., ASNPC and NS_max_) were lower in the pot trial in comparison with the field trial (Figure 1).

### Trait Correlations and Heritability

**Figure 2:**
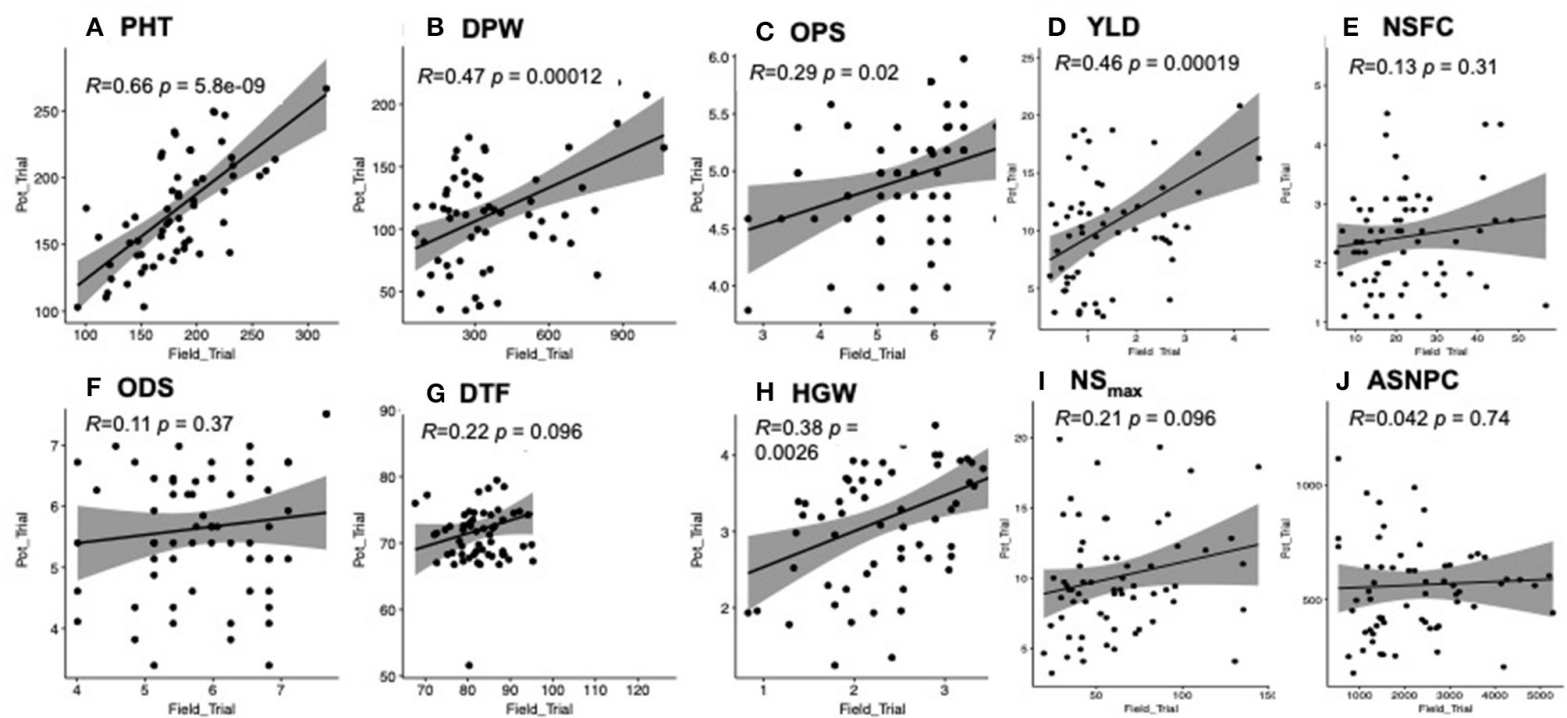
**(A-J)** Correlation of traits between field and pot trials. Significant correlations were observed for plant height (PH), dry panicle weight (DPW), overall pest score (OPS), yield (YLD), and 100-grain weight (HGW) at *P* < 0.05.

We observed positive and significant correlations between the yield-related data collected from the field and pot trials for all traits (i.e., PH, DPW, YLD, and HGW) except DTF (Figure 2). There was a weak non-significant correlation between the two trials for all the biotic stress-related traits (i.e., NSFC, ASNPC, NS_max_, and ODS) except OPS (Figure 2).

We also looked at trait correlations within each trial and recorded more significant trait correlations (*P* < 0.05) in the pot trial (21) than in the field trial (13) (Supplementary Table 1). Yield-related traits (i.e., HGW, YLD, DTF, and PH) were negatively correlated with ASNPC, NS_max_, NSFC, ODS, and OPS, in both field and pot trials, although the correlation was largely non-significant (Supplementary Table 1). The highest positive significant correlations in the field trial were recorded between ASNPC and NS_max_ (*r* = 0.83; *P* < 0.001), ASNPC and NSFC (*r* = 0.77; *P* < 0.001), and NS_max_ and NSFC (*r* = 0.80; *P* < 0.001). The same traits were also highly positively correlated in the pot trial with comparable correlation values of *r* = 0.84 (i.e., ASNPC and NS_max_) and *r* = 0.73 (i.e., NSFC and ASNPC), except for the correlation between NSFC and NS_max_ (*r* = 0.55).

**Figure 3:**
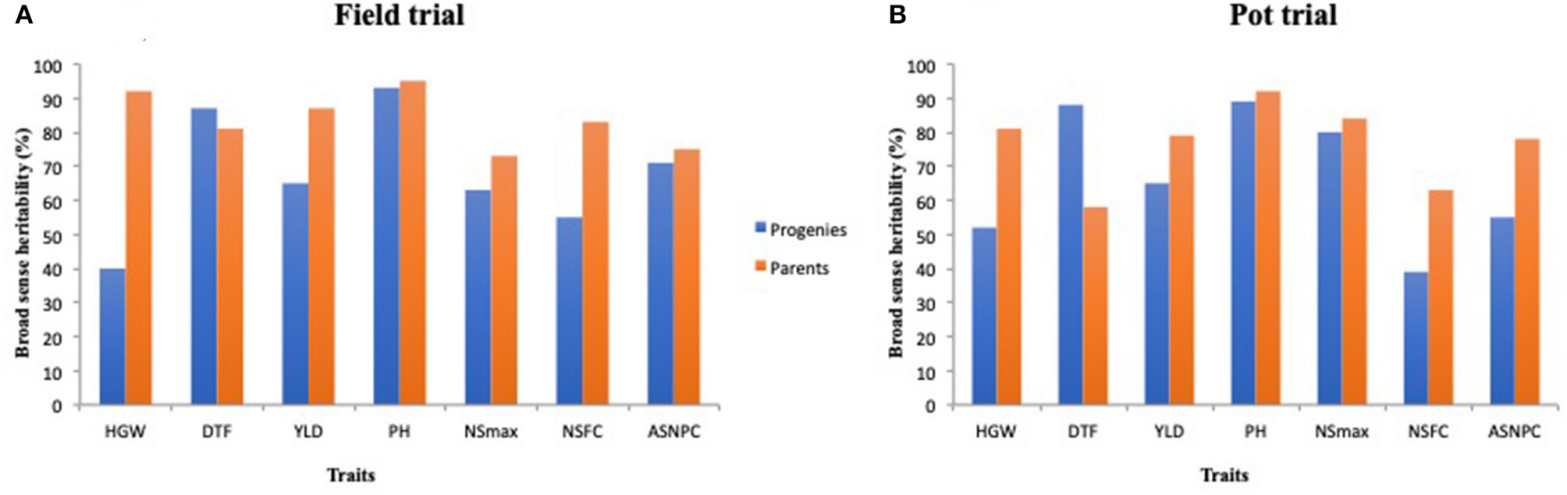
Broad-sense heritability estimates for select traits among parents and progenies evaluated under the field trial **(A)** and pot trial **(B)**.

Parents recorded high heritability values for all traits except DTF under the pot trial (Figure 3, Supplementary Table 1). Progenies displayed relatively lower heritability values for most traits in comparison with the parents, except for DTF in both environments (Figure 3).

### Agronomic Performance of the Germplasm Under Field and Pot Trials

**Figure 4:**
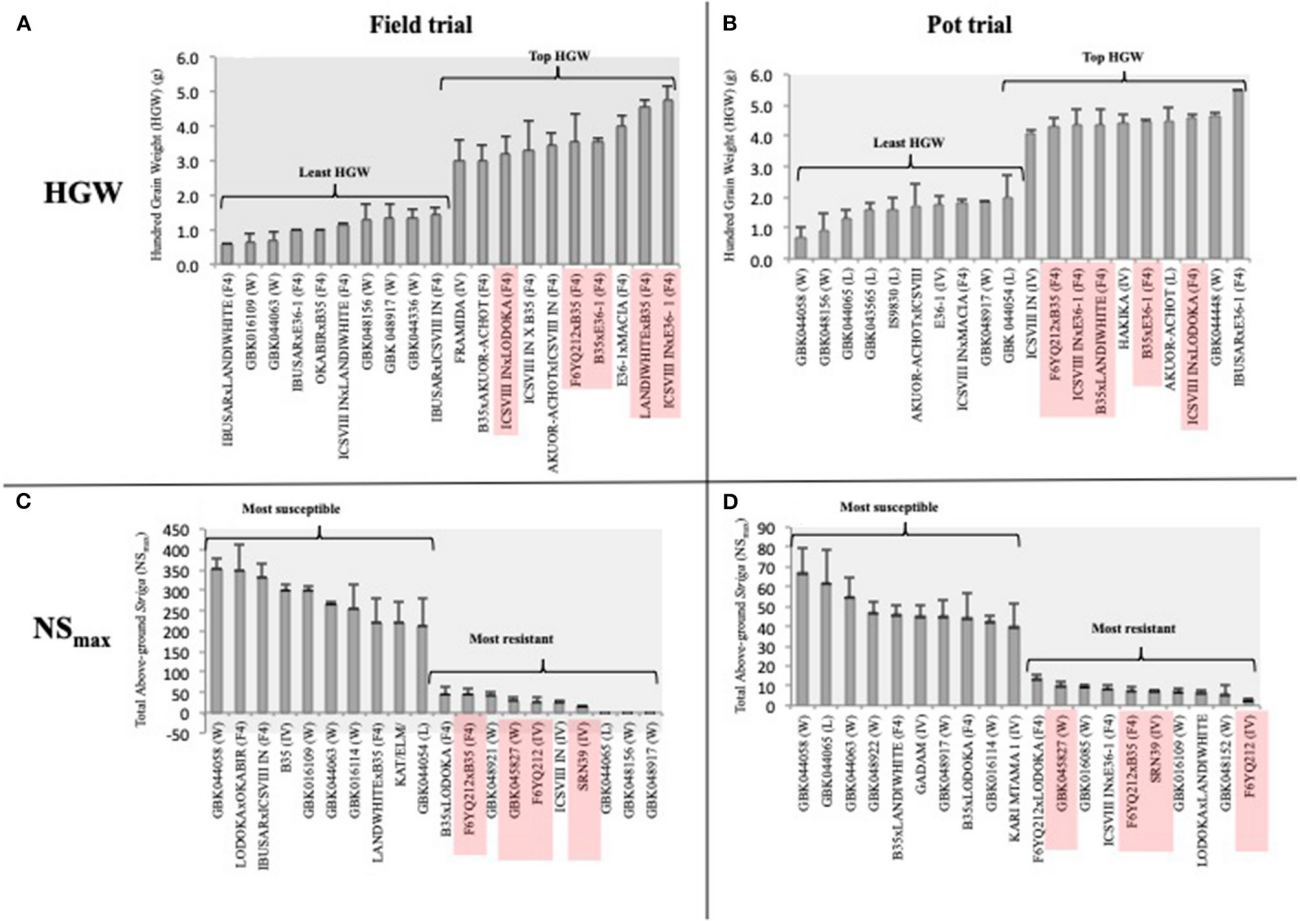
The top 10 best and worst performers for HGW and *Striga* (NS_max_) in the field **(A,C)** and pot trial **(B,D)**, respectively. Genotypes with consistent performance across the two environments are highlighted in red.

We used HGW rather than YLD to compare the yield performance of genotypes between the two trials, given the differences in the trial conditions that would bias the total yield comparisons. Of note, 9 and 6 out of the 10 genotypes with the highest HGW in the field and pot trials, respectively, were F_4_ progenies (Figures 4A,B). Five (ICSVIII IN × E36-1, LANDIWHITE × B35, B35 × E36-1, F6YQ212 × B35, and ICSVIII IN × LODOKA) of the F_4_ progenies with the highest HGW were common in both trials (Figures 4A,B), revealing their potential stability for the trait. FRAMIDA and HAKIKA, which are both *Striga*-resistant and improved varieties, were the only resistant checks among the top 10 genotypes recording the highest HGW in the field and pot trials, respectively (Figures 4A,B). A landrace, AKUOR-ACHOT, and a wild accession, GBK044448, were also among the top 10 genotypes with high HGW of 4.5 and 4.7 g, respectively, in the pot trial (Figure 4B) but not in the field trial (Supplementary Table 1). Most of the genotypes with the lowest HGW were wild accessions or landraces in both trials, although some improved varieties (i.e., E36-1 in pot trial) and F_4_ progenies also fell into this category (Figures 4A,B).

### Response of Genotypes to *Striga* Under Field and Pot Trials

Despite the poor correlation of *Striga*-related traits between pot and field trials, we observed stability in the performance of four (F6YQ212, SRN39, F6YQ212xB35, and GBK045827) out of the 10 top *Striga*-resistant genotypes in both field and pot trials (Figures 4C,D) according to their NS_max_ ranking (Figures 4C,D). SRN39, a stable source of resistance to *Striga*, was the only resistant check among the topmost resistant genotypes in both trials (Figures 4C,D). Although the F_4_ progenies were the dominant best performers for HGW, they were the minority genotypes among the top 10 most *Striga*-resistant genotypes in both field and pot trials. F6YQ212, which showed resistance to *Striga* in both trials, was the common parent in two out of the three F_4_ progenies with resistance to *Striga*, suggesting it would be a good source of stable *Striga* resistance for future crosses. B35, a drought-tolerant variety, which was among the top 10 most *Striga*-susceptible genotypes in the field trial, was the common parent in two out of the three top *Striga*-resistant progenies in both trials (Figures 4C,D). The only genotype that was recorded among the top performers in both trials for HGW and *Striga* resistance (NS_max_) was a cross between the *Striga*-resistant variety, F6YQ212, and the drought-tolerant variety, B35 (Figure 4).

Three genotypes that included two wild (GBK048156 and GBK048917) and a landrace (GBK044065) recorded no *Striga* germination in the field trial but supported the germination of significant amounts of *Striga* in the pot trial (Supplementary Table 1). A wild accession, GBK044058, was the most susceptible genotype to *Striga* in both trials. All genotypes tested for the *Striga* germination in the pot trial supported the germination of at least three *Striga* plants in at least one replicate (Supplementary Table 1). However, genotype GBK016109, which showed comparable resistance to *Striga* as SRN39 in the pot trial, was completely devastated in the field trial recording one of the worst performers (Figures 4C,D).

### Genotype Relatedness and Confirmed Hybridity

**Figure 5:**
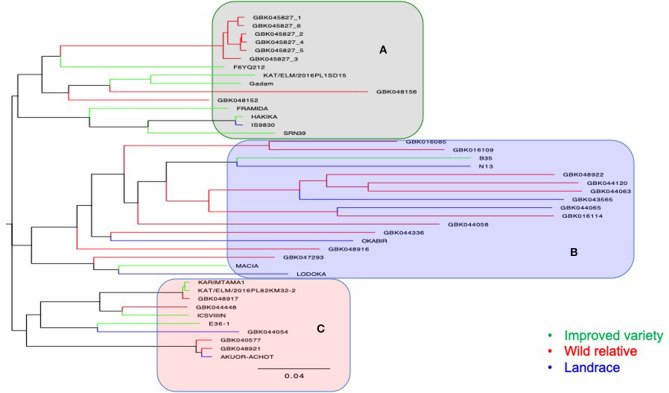
A neighbor-joining dendrogram showing genetic relatedness of 37 accessions that comprised of 17 wild accessions, 8 landraces, and 12 improved varieties. The main clusters generated are highlighted in gray **(A)**, light blue **(B)**, and pink **(C)**.

A total of 26,291 raw SNPs were generated from DArT-seq of 64 genotypes (i.e., 17 wild, 8 landraces, 12 improved varieties, and 27 F_4_ progenies), of which 7,038 SNPs were retained after filtering. The NJ dendrogram resulted in three major clusters (Figure 5). The first cluster (A) (Figure 5) comprised of *Striga*-resistant genotypes including four resistant checks, namely, IS9830, SRN39, FRAMIDA, and HAKIKA. Other genotypes in cluster A were recorded as resistant to *Striga* in this study such as GBK048156 (field trial), GBK048152 (pot trial), F6YQ212 (field trial and pot trial), and GBK045827 (field trial and pot trial). The only susceptible genotype in this cluster was the improved variety KAT/ELM/2016PL1SD15. Cluster B comprised mostly of wild accessions and landraces. Two improved varieties, namely, B35 and MACIA, were also in cluster B, but different subclusters. The only *Striga*-resistant check in this cluster was N13, which was grouped in the same subcluster with the staygreen genotype, B35. Both B35 and N13 are known to have wild pedigrees.

Two wild genotypes (GBK016109 and GBK016085), which were among the top most *Striga*-resistant lines in the pot trial, and one landrace (GBK044065), which was among the most resistant lines in the field trial, were also grouped in cluster B (Figure 5). Cluster C was composed of four improved varieties, two landraces, and four wild accessions. Two genotypes (GBK048917 and ICSV III IN), which were also among the most resistant to *Striga* in the field trial, were also grouped under cluster C. Genotype E36-1, a well-known drought-tolerant (staygreen) material, was also grouped in cluster C alongside a landrace, GBK044054, which recorded one of the highest NS_max_ in the field trial (Figure 4C).

Crosses involving parental lines IBUSAR and LANDIWHITE were not included in the hybridity analysis as both parents failed to germinate in both trials. Hybridity of 16 F_4_ progenies was confirmed using biallelic SNP markers ranging from 1,204 to 2,868 that had been called from DArT-seq (Table 4). The highest proportion (22–46%) of heterozygous alleles were recorded in the progenies of the cross B35 × E36-1, while the lowest (<1%) were recorded in the crosses OKABIR × AKUOR-ACHOT and LODOKA × OKABIR. B35 and E36-1 are the improved varieties derived from wild backgrounds, while LODOKA and AKUOR-ACHOT are landraces.

**Table 4:** Confirmation of hybridity among the fourth filial generation (F_4_) progenies using *Striga* number progress (SNP) markers.

**Cross**	**Plants tested**	**Average no. of biallelic markers**	**Proportion of heterozygous alleles (%)**
1. B35 × ICSVIII IN	6	2,806	<1–33
2. B35 × F6YQ212	6	2,774	15–43
3. B35 × AKUOR-ACHOT	6	2,699	1–4.5
4. E36-1 × MACIA	6	1,485	<1–29
5. F6YQ212 × LODOKA	5	1,947	2–13
6. B35 × ICSVIII IN	6	2,806	<1–33
7. B35 × E36-1	6	2,868	22–46
8. B35 × LODOKA	6	2,467	9–25
9. F6YQ212 × B35	5	2,695	<1–10
10. ICSVIII IN × E36-1	6	1,313	14–33
11. LODOKA × ICSVIII IN	6	1,825	<1–15
12. OKABIR × ICSVIII IN	6	2,339	<1–2
13. OKABIR × AKUOR-ACHOT	6	2,174	<1
14. LODOKA × OKABIR	6	2,357	<1
15. ICSVIII IN × MACIA	6	1,204	10–17
16. ICSVIII IN × LODOKA	4	1,764	4–13

### Residual Heterosis for Yield and Resistance to *Striga* in the F_4_ Progenies

A complete record of the MP and residual heterosis values in the field and pot trials for HGW and NS_max_ is shown in Supplementary Table 2. Table 5 shows the residual heterosis values for HGW in each of the crosses in the field and pot trials ranked from the top to the lowest. Both the highest residual heterosis and the inbreeding depression for HGW were recorded in the field at 89.78% and −59.48%, respectively. LODOKA, a drought-tolerant landrace, was the common parent in the crosses with the highest residual heterosis in both trials (Table 5). Four (i.e., AKUOR-ACHOT × ICSVIII IN, B35 × AKUOR-ACHOT, ICSVIII IN × MACIA, LODOKA × ICSVIII IN) out of six crosses that recorded the inbreeding depression for HGW were consistent in both the field and pot trials (Table 5), suggesting they would be poor candidates for yield-related traits. Crosses involving ICSVIII IN, an improved variety, revealed some of the highest inbreeding depression for HGW in both field and pot trials (Table 5). In some cases, the highest residual heterosis recorded for HGW (86.19% for F6YQ212 × B35 and 52.94% for B35 × LODOKA) also corresponded to a low *Striga* count of −65.13 and −79.20% (Tables 5, 6), making these crosses good candidates for the future development of *Striga*-tolerant, high-yielding varieties.

**Table 5:** Proportion of residual heterosis at F_4_ for 100-grain weight (HGW) for the pot and field trials.

**Field**		**Pot**	
**Crosses**	**HGW (%)**	**Crosses**	**HGW (%)**
1. F6YQ212 × LODOKA	89.78	1. LODOKA × OKABIR	79.33
2. F6YQ212 × B35	86.19	2. E36-1 × MACIA	46.52
3. ICSVIII_IN × LODOKA	77.61	3. OKABIR × B35	31.03
4. B35 × LODOKA	52.94	4. B35 × E36-1	21.44
5. LODOKA × OKABIR	47.33	5. B35 × ICSVIII_IN	20.99
6. OKABIR × B35	41.44	6. ICSVIII_IN × E36-1	15.77
7. OKABIR × ICSVIII_IN	39.30	7. F6YQ212 × LODOKA	11.31
8. ICSVIII_IN × B35	30.25	8. OKABIR × ICSVIII_IN	10.14
9. B35 × F6YQ212	25.83	9. B35 × LODOKA	8.06
10. B35 × E36-1	24.53	10. OKABIR × AKUOR-ACHOT	4.52
11. E36-1 × MACIA	19.76	11. F6YQ212 × B35	3.33
12. ICSVIII_IN × E36-1	18.33	12. ICSVIII_IN × LODOKA	2.67
13. AKUOR-ACHOT × ICSVIII_IN	−0.50	13. B35xF6YQ212	−8.33
14. B35 × AKUOR-ACHOT	−6.65	14. B35 × AKUOR-ACHOT	−21.58
15. B35 × ICSVIII_IN	−26.31	15. LODOKA × ICSVIII_IN	−22.32
16. ICSVIII_IN × MACIA	−27.05	16. ICSVIII_IN × B35	−37.46
17. LODOKA × ICSVIII_IN	−28.36	17. ICSVIII_IN × MACIA	−38.82
18. OKABIR × AKUOR-ACHOT	−59.48	18. AKUOR-ACHOT × ICSVIII_IN	−48.51

**Table 6:** Proportion of residual heterosis at F_4_ for maximum above-ground *Striga* (NS_max_) for the pot and field trials.

**Field**		**Pot**	
**Crosses**	NS_**max**_ (%)^*^	**Crosses**	NS_**max**_ (%)^*^
1. B35 × LODOKA	−79.2	1. F6YQ212 × B35	−53.52
2. B35 × ICSVIII_IN	−71.21	2. ICSVIII_IN × B35	−45.44
3. B35 × AKUOR-ACHOT	−66.02	3. OKABIR × B35	−32.24
4. F6YQ212 × B35	−65.13	4. ICSVIII_IN × E36-1	−29.39
5. OKABIR × B35	−51.03	5. B35 × F6YQ212	−6.91
6. B35 × F6YQ212	−51.01	6. B35 × ICSVIII_IN	−5.4
7. ICSVIII_IN × B35	−49.36	7. LODOKA × OKABIR	−2.27
8. B35 × E36-1	−31.89	8. OKABIR × AKUOR-ACHOT	−1.81
9. OKABIR × AKUOR-ACHOT	−17.53	9. F6YQ212 × LODOKA	−0.12
10. ICSVIII_IN × LODOKA	15.48	10. B35 × AKUOR-ACHOT	1.57
11. ICSVIII_IN × E36-1	35.85	11. AKUOR-ACHOT × ICSVIII_IN	4.37
12. OKABIR × ICSVIII_IN	43.09	12. E36-1 × MACIA	21
13. F6YQ212 × LODOKA	62.09	13. ICSVIII_IN × MACIA	24.4
14. LODOKA × ICSVIII_IN	63.73	14. ICSVIII_IN × LODOKA	26.28
15. E36-1 × MACIA	75.88	15. B35 × E36-1	39.64
16. AKUOR-ACHOT × ICSVIII_IN	115.14	16. B35 × LODOKA	57.89
17. LODOKA × OKABIR	144.68	17. OKABIR × ICSVIII_IN	62.88
18. ICSVIII_IN × MACIA	289.77	18. LODOKA × ICSVIII_IN	136.78

* *Values with negative percentage are the most desirable as they show a lower number of Striga plants than that of the midparent*.

Table 6 shows the residual heterosis values for NS_max_ in each of the crosses in the field and pot trials. Crosses involving B35, a drought-tolerant improved variety, had some of the highest inbreeding depression values for NS_max_, which is desirable as it resulted in more resistance to *Striga* (Table 6). Like in HGW, crosses involving ICSVIII IN performed worse than the MP for their resistance to *Striga*, indicating that this parent may not be a good choice for improving either yield or *Striga* resistance (Table 6).

## Discussion

### Identification of Diverse High-Yielding *Striga*-Resistant Genotypes

The aim of this study was to identify new sources of *Striga* resistance in comparison with the conventional sources. The data collected found credible evidence that genotypes that were resistant to *Striga* had significantly lower ASNPC and NS_max_-values in both trials. These parameters were considered alongside grain yield for the effective selection of superior *Striga*-resistant, as well as *Striga*-tolerant genotypes as recommended by Rodenburg et al. (2005). We identified three genotypes (F6YQ212, GBK045827, and F6YQ212 × B35) together with one check (SRN39) that were consistent in their response to *Striga* across both trials. SRN39 is known to harbor pre-attachment resistance that results in the production of a low germination stimulant, orobanchol (Satish et al., 2012; Mohemed et al., 2016). However, SRN39 is not high yielding and, therefore, not preferred by farmers and has been used mainly as a donor of *Striga* resistance to other improved varieties. Of specific interest is the cross between improved varieties, F6YQ212 × B35, which recorded consistency in resistance to *Striga* and was also high yielding. This particular cross is likely to perform well in the fields of farmers and would be a good genotype to advance for field trials.

The wild accession, GBK045827, which also showed consistency in *Striga* resistance across the two trials, did not only group together with F6YQ212 but also recorded comparable performance with F6YQ212 in both experiments. This observation strongly suggests that the resistance observed in F6YQ212 may have been introgressed from GBK045827. We know that *Striga* resistance is more abundant among wild relatives (Rich et al., 2004; Mbuvi et al., 2017), which tend to cross-pollinate with cultivated genotypes in open fields (Ohadi et al., 2017). Both F6YQ212 and GBK045827 clustered in group A with four resistant checks (FRAMIDA, HAKIKA, IS9830, and SRN39) but in a different subclade. This pattern of clustering suggests a narrow genetic base for *Striga*-resistant sources that are currently being used in breeding programs in Eastern Africa (Mohamed et al., 2010), except for N13. Genotype F6YQ212, therefore, provides a good alternative source of resistance as it was grouped in a different subclade. F6YQ212 has been previously screened for response to grain storage pests (Mwenda, 2019) but not for its resistance to *Striga*. Future studies will therefore need to determine the mode of resistance in F6YQ212, as well as in GBK045827. The mechanism of resistance in FRAMIDA, IS9830, and SRN39 is reported to be low germination stimulation (Haussmann et al., 2000b; Mohamed et al., 2010; Gobena et al., 2017), which is the most widely studied mechanism of resistance to *Striga* in sorghum. The only other resistant check that clustered differently was N13, a durra sorghum from India, which is known to stimulate *Striga* germination but forms a mechanical barrier to *Striga* penetration (Maiti et al., 1984; Mohemed et al., 2016; Mbuvi et al., 2017). Genotype N13 grouped together with B35, a drought-tolerant variety, which has its origins in Ethiopia (Ochieng et al., 2020).

### Screening for *Striga* Resistance in Pot and Field Trial Conditions

The trials made use of an existing *Striga*-infested field that was supplemented with artificial *Striga* inoculation, as well as a pot trial with artificial *Striga* inoculation to represent a second environment. One of the major challenges of undertaking reliable field trials using *Striga*-infested fields is the lack of homogeneity of *Striga* infections across different points of the field. This is especially due to the outcrossing nature of *Striga* and the tendency of its seeds to remain dormant in the soil for up to 20 years (Teka, 2014). In our investigation, both field trials and inoculated pots were used to take care of natural infection conditions, as well as enhance uniformity of infection. There are a number of successful *Striga* studies that used both field and pot experiments together with supplemented artificial inoculation in the same way (Kountche et al., 2013; Rodenburg et al., 2015; Midega et al., 2016; Abate et al., 2017). Other studies also made use of pot experiments as *Striga*-free control (Samejima et al., 2016), or to enable the isolation of root exudates (Jamil et al., 2011; Hooper et al., 2015). The low correlation reported here between field and pot experiments for *Striga*-related traits has also been observed in other experiments (Haussmann et al., 2000a), suggesting that the use of pots for *Striga* screening should be completely discouraged.

Several factors could explain the observed differences in the pot and field experiments. First, the *Striga* infestation levels were significantly higher in the field trial than in the pot experiments, in which *Striga*-free soil was used before the addition of a standard amount of *Striga* in each pot. Second, it is likely that the field trial had a mixture of biotypes that had been accumulated over the years before the supplemented inoculation. Third, the *Striga* in the field trial was established exclusively under rainfed conditions, whereas the pot experiment was regularly watered to ensure sufficient moisture was maintained throughout the experiment. Furthermore, there could be rhizosphere differences that would affect the stimulation of *Striga* germination (Miché et al., 2000). Variable response to *Striga* under different test conditions has been observed in previous studies (Rao, 1984; Haussmann et al., 2000a).

Haussmann et al. (2004) hypothesized that abiotic stress, followed by ethylene production by microorganisms in the soil, could be responsible for the observed differences. In this study, drought stress could have been a factor in the field trial experiment, which was purely dependent on rain. Drought has been shown to induce strigolactone production in the roots (Haider et al., 2018), which in turn induces the germination of *Striga* (Cardoso et al., 1994). A significant decrease in rainfall in Kenya has been reported over the last four decades (Ayugi et al., 2016), suggesting that rainfed crops are highly likely to be exposed to drought during their growth periods. Rainfall data (not shown) from Alupe station during the growing season further confirmed the variability of rainfall that could have led to the exposure of the crops to drought. Nonetheless, we observed a number of genotypes showing consistency in their performance between the two trials for *Striga*-related traits.

### Mechanism of Resistance to *Striga* in N13

Host plant resistance to *Striga* has been defined as the ability of the plant to reduce or prevent infection (Shew and Shew, 1994) through pre- and post-attachment mechanisms (Yoder and Scholes, 2010; Rodenburg et al., 2016). Tolerance refers to the extent to which effects of infection on the host plant are mitigated (Caldwell et al., 1958; Rodenburg et al., 2016). In this evaluation, N13 was not among the most resistant genotypes in both experiments. A study by Rodenburg et al. (2005) that incorporated N13, SRN39, IS9830, and FRAMIDA among other genotypes reported N13 as the most resistant genotype in the trial with ASNPC and NS_max_-values significantly lower than all the other genotypes in the trial. Our results may suggest a possible breakdown of mechanical resistance that was initially recorded in N13, or potential contamination of seed source that may have led to the reduction in the resistance originally observed. However, these theories will need to be further investigated.

Although the genetic architecture of mechanical resistance to *Striga* in sorghum is yet to be understood, previous studies suggest that it is complex. QTL mapping studies in recombinant inbred populations using N13 as the resistant parent reported 11 and 9 QTLs in two different environments (Haussmann et al., 2004). The durability of mechanical resistance will therefore depend on the factors at play, which may range from cell wall thickening, lignification, and silica deposition (Maiti et al., 1984). A recent study of *Striga* resistance in rice reported that enhanced lignin deposition and maintenance of the structural integrity of lignin polymers deposited at the infection site are crucial for post-attachment resistance against *S. hermonthica* (Mutuku et al., 2019). Similar studies will be necessary in sorghum to enhance our understanding of both pre- and post-attachment resistance to *Striga*. Such an outcome will enable the pyramiding of genes responsible for both pre- and post-attachment resistance in order to enhance the durability of resistance in the fields of farmers.

### Drought Tolerance and Yield Stability Under *Striga* Infestation

A majority of the best yielding (most tolerant) genotypes were derived from crosses. However, the most consistent performance among these top-performing crosses was observed when crosses were made with any of the drought-tolerant genotypes, such as LODOKA, B35, and E36-1. B35 and E36-1 are drought-tolerant improved varieties that have been used for decades in the region and globally, while LODOKA is a drought-tolerant landrace. The superior and consistent agronomic performance of crosses involving drought-tolerant genotypes under *Striga* conditions was not surprising. Both drought stress and *S. hermonthica* infestation result in the production of abscisic acid (ABA) (Frost et al., 1997; Sah et al., 2016), which triggers stomatal closure (Kim et al., 2010). However, drought-tolerant sorghum genotypes have been shown to adapt to drought stress by preventing excessive ABA responses (Varoquaux et al., 2019). Such an adaptation response of drought-tolerant sorghum would also benefit their response to *S. hermonthica* and enhance the production of photosynthates to sustain plant growth and development in the presence of both stresses. A recent study in maize reported up to 19% increase in yield under stress in hybrids simultaneously expressing drought tolerance and *S. hermonthica* resistance as compared with those expressing only one of the traits (Menkir et al., 2020). Similar studies will need to be undertaken in sorghum to fully establish the interaction of drought and *S. hermonthica* stresses.

The older sources of drought tolerance will, however, need to be replaced with new superior performing genotypes such as F6YQ212 × B35 and ICSVIII IN × LODOKA identified in the current study. OKABIR, AKUOR-ACHOT, and LODOKA are landraces, which have also been recently reported to perform better than B35 and E36-1 under drought conditions (Ochieng et al., 2020). While LODOKA clustered with MACIA, both OKABIR and AKUOR-ACHOT appeared to be distantly related to MACIA, B35, and E36-1 and will therefore be good alternative sources of both drought and *Striga* tolerance. Better still, more focused introgression of *Striga* resistance and drought tolerance into farmer-preferred varieties should be planned to ensure better replacement of some of these old varieties.

### Molecular Breeding for *Striga* Resistance and Tolerance

The demonstrated residual heterosis in HGW and NS_max_ at F_4_ is great news for breeding programs as it shows the huge potential of enhancing the performance of varieties in response to *Striga* through improved genetics. Given the high variation in the ecotypes of *Striga* across different environments, the best breeding strategy would be genomic selection (GS) (Goddard, 2009). Our results provide a good basis for designing a GS strategy for developing *Striga*- and drought-tolerant sorghum varieties that will be suitable for the harsh environments typical of *Striga*-endemic ecologies. The available genomic resources in sorghum public databases will only enhance the ease with which GS is implemented in sorghum.

The DArT-seq technology (Sansaloni et al., 2011) that was used to characterize the germplasm proved to be a reliable and cost-effective technology for diversity analysis and confirming hybridity. While there are several studies reporting the use of DArT-seq for diversity analysis in sorghum (Kotla et al., 2019; Allan et al., 2020; Mengistu et al., 2020), this study is the first to use DArT-seq for hybridity testing. The unique SNP markers from this study will be useful for GS and for incorporation into marker panels that aim at the identification of successful hybrids from new crosses involving any of the parents. Future studies will also need to establish the specific molecular markers associated with new sources of resistance to *Striga* through genome-wide association studies (GWAS) or the characterization of biparental populations.

## Data Availability Statement

The datasets presented in this study can be found in online repositories. The names of the repository/repositories and accession number(s) can be found in the article/Supplementary Material.

## Author Contributions

KN and DO conceived the project, designed and supervised the experiment, and drafted the manuscript. NM did both the field and laboratory experiments and drafted the manuscript. EM provided some of the germplasm and supervised the fieldwork and data collection. WC supervised the fieldwork and data collection. MA supported the laboratory work and did part of the data analysis. DN and LW provided the genebank material and supervised the work, respectively. All authors approved the final version of the manuscript.

## Conflict of Interest

The authors declare that the research was conducted in the absence of any commercial or financial relationships that could be construed as a potential conflict of interest.
